# Zinc Transporters, Mechanisms of Action and Therapeutic Utility: Implications for Type 2 Diabetes Mellitus

**DOI:** 10.1155/2012/173712

**Published:** 2012-12-12

**Authors:** Stephen A. Myers, Alex Nield, Mark Myers

**Affiliations:** ^1^School of Health Sciences, University of Ballarat, University Drive, Mount Helen, VIC 3350, Australia; ^2^Collaborative Research Network, University of Ballarat, Mount Helen, VIC 3350, Australia

## Abstract

Zinc is an essential trace element that plays a vital role in maintaining many biological processes and cellular homeostasis. Dysfunctional zinc signaling is associated with a number of chronic disease states including cancer, cardiovascular disease, Alzheimer's disease, and diabetes. Cellular homeostasis requires mechanisms that tightly control the uptake, storage, and distribution of zinc. This is achieved through the coordinated actions of zinc transporters and metallothioneins. Evidence on the role of these proteins in type 2 diabetes mellitus (T2DM) is now emerging. Zinc plays a key role in the synthesis, secretion and action of insulin in both physiological and pathophysiological states. Moreover, recent studies highlight zinc's dynamic role as a “cellular second messenger” in the control of insulin signaling and glucose homeostasis. This suggests that zinc plays an unidentified role as a novel second messenger that augments insulin activity. This previously unexplored concept would raise a whole new area of research into the pathophysiology of insulin resistance and introduce a new class of drug target with utility for diabetes pharmacotherapy.

## 1. Introduction

Type 2 diabetes mellitus (T2DM) is a progressive and debilitating disorder characterized by a loss of glycaemic control and metabolic homeostasis through the deterioration of beta cell function [1] and relative insulin deficiency and insulin resistance [2] in peripheral tissues including skeletal muscle, adipose, and liver [3]. The prevalence of this metabolic disorder is expected to increase from 171 million people in 2000 to 366 million in 2030, predominately due to changes in nutrition, reduced physical activity, and obesity [4]. Complications arising from T2DM include renal failure [5], blindness [6], and dyslipidemia [7], and it is widely accepted to be a fundamental and foremost basis for cardiovascular disease [8]. Given this projection, T2DM is becoming a leading cause of morbidity and mortality that results in a significant reduction in quality of life and life expectancy [9]. 

Recent developments in our understanding of T2DM have been heightened by the potential relevance of dysfunctional zinc signaling in this disease. Indeed, the early seminal discovery that insulin crystals contain zinc [10] facilitated a supportive role for this cation in diabetes. Zinc is an essential trace element that is indispensable for its role in maintaining normal physiological function and cellular homeostasis [11, 12]. Disturbances in zinc homeostasis have been observed in diabetes [9, 13–18] and several other pathologies including cancer [19, 20], autoimmune disease [21, 22], cardiovascular disease [11, 13, 23, 24], and Alzheimer's disease [25, 26]. 

Zinc has three major biological roles: catalytic, structural and regulatory [27]. The catalytic and structural role of zinc is well recognized, and there are many noteworthy reviews on these functions ([28–31] and references therein). For example, zinc is a structural constituent in numerous proteins, including growth factors, cytokines, receptors, enzymes, and transcription factors belonging to cellular signaling pathways [30]. Moreover, it is implicated in numerous cellular processes as a cofactor for an estimated 3000 human proteins including enzymes, nuclear factors, and hormones [32]. 

**Figure 1:**
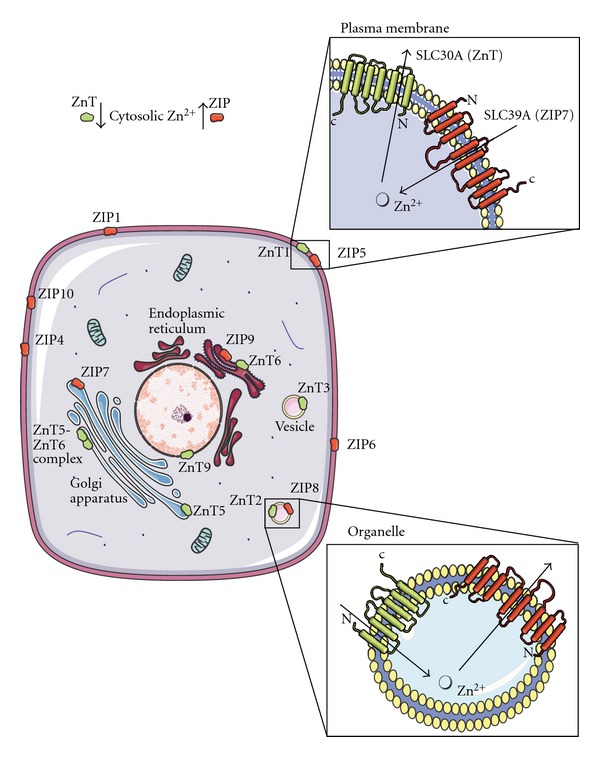
*Subcellular localization and direction of transport of the zinc transporter families, ZnT and ZIP*. Arrows show the direction of zinc mobilization for the ZnT (green) and ZIP (red) proteins. A net gain in cytosolic zinc is achieved by the transportation of zinc from the extracellular region and organelles such as the endoplasmic reticulum (ER) and Golgi apparatus by the ZIP transporters. Cytosolic zinc is mobilized into early secretory compartments such as the ER and Golgi apparatus by the ZnT transporters. Figures were produced using Servier Medical Art, http://www.servier.com/.

Homeostatic mechanisms that modulate zinc absorption, distribution, cellular uptake, and excretion [27] are vital for maintaining cellular function. Zinc's fundamental and diverse role in many cellular processes requires that zinc delivery to tissues and cells, intracellular availability, and intracellular distribution are tightly controlled [33]. These processes are maintained through the coordinated orchestration of a diverse family of transport proteins that modulate the uptake, efflux, and compartmentalization of zinc [33]. In this context, 4 metallothioneins (MTs), 14 zinc importers (SLC39/ZIPs), and 10 zinc exporters (SLC30/ZnTs) have been described in mammals [34]. (Human zinc transporters are defined as SLC30A and SLC39A while rodent transporters are designated Slc30a and Slc39a. For brevity, species designations are not used in this paper and transporters will be defined as SLC39A/ZIP and SLC30A/ZnT. Present terminology and accession numbers can be obtained through the GenBank database, http://www.ncbi.nlm.nih.gov/genbank). The MTs are the major zinc-binding proteins in mammalian systems that play an important regulatory role in zinc uptake, storage, release, and distribution [35, 36]. The zinc transporters are essential for preserving zinc homeostasis where the ZIP transporters contribute to a net increase in cytosolic zinc while the ZnTs cause a net decrease in cytosolic zinc [30] (Figure 1). 

In the context of T2DM, essential dietary zinc and proteins that modulate zinc metabolism play a major role in metabolic homeostasis in peripheral tissues that respond to insulin. However, our understanding of the relationships between zinc transport, cellular zinc signaling, and T2DM is limited. Accordingly, this paper aims to delineate the role of zinc transporter systems, their mechanisms of action, and possible roles in disease with a particular focus on T2DM. 

## 2. Zinc and Zinc Transporters: Regulation, ****Signaling, and Cellular Mechanisms 

There is approximately 2–4 g of zinc in the human body but the concentration of the mobile pool of zinc ions in plasma is only 12–16 *μ*M [9]. Given the low plasma concentration of zinc and its importance in cellular processes, it is essential that the availability and distribution of “free zinc” (“free zinc” is an operative term that is used to differentiate the zinc that is implicated in signaling transduction from that of zinc tightly bound to protein and thermodynamically unavailable)is tightly controlled [37]. Diet replenishes approximately 1% of the total body zinc lost by intestinal excretion in humans which is principally achieved by intestinal absorption [33]. Zinc delivery to tissues and cells and its intracellular distribution is also tightly regulated by a family of proteins that control uptake, efflux, and compartmentalization of zinc. In mammals, this is accomplished by an array of zinc transporters encoded by at least 27 genes from 3 families. These are the SLC30 (CDF/ZnT: vertebrate cation diffusion facilitator family proteins), SLC39 (ZIP: Zrt-,Irt-like Proteins) solute transporters, and the zinc-sensitizing MTs [18, 20, 34, 38–40] (Figure 1). The MTs play an important regulatory role in zinc uptake, storage, distribution, and release [29, 36]. The ZIP family members facilitate the influx of zinc into the cytosol from the outside of the cells and from the lumen of intracellular compartments, while the ZnT family members enable the transport of zinc out of the cytosol into the lumen of intracellular organelles or to the outside of the cell [27, 34, 40, 41]. Both ZIP and ZnT transporters work in an opposite but coordinated way to maintain cellular zinc homeostasis. Although the tissue distribution of the ZIP and ZnT family has been investigated in detail [42] (Table 1), in addition to differential expression based on gender [43], their mechanisms of action are still not well understood.

### 2.1. The SLC39/ZIP Family

ZIP transporters were first identified as “Zrt-, Irt-like Proteins” following their identification in yeast *Saccharomyces cerevisiae* (Zrt; zinc regulated transporter) and their similarity to the Fe(II) transporter Irt1 protein from the plant *Arabidopsis thaliana* [44, 45]. In these studies, Zhao and Eide [44] showed that overexpression of Zrt1 in yeast cells increased high affinity uptake of ^65^Zn and that this was reduced in Zrt1 mutant cells resulting in poor growth in zinc-limited media. Since their discovery, the ZIP family of proteins has grown to more than 100 members including those from insects, bacteria, nematodes and mammals [38, 46]. In mammals, members of this family are designated SLC39 for solute carrier [40]. Most ZIP transporters are predicted to have eight transmembrane (TMD) domains and similar predicted topologies (Figure 1). Characteristics of this topology have been confirmed for yeast [47]. Many of the family members have a long loop region between TMD III and IV that frequently contains a histidine-rich region [(HX_*n*_, *n* = 3 to 5)] [40] that is suggested to be a putative zinc-binding domain [48]. The majority of ZIP proteins share a similar predicted topology where both the N and C-termini are extracytoplasmic [40]. A key feature of ZIP transporters is that they facilitate the influx of zinc into the cytosol from the extracellular space and from the lumen of intracellular compartments into the cytoplasm [30]. 

### 2.2. SLC30/ZnT Family

The first ZnT protein (termed ZnT1) was described by Palmiter and Findley [49]. ZnT1 was isolated from a rat kidney cDNA library and shown to restore zinc resistance when transfected into a zinc sensitive baby kidney hamster (BHK) cell line under high levels of extracellular zinc [49]. To date, the mammalian SLC30 family code for ten zinc transporters (ZnTs), ZnT1-10 [50]. The ZnT family of zinc transporters enables the transport of zinc out of the cytosol into the lumen of intracellular organelles or to the outside of the cell [27, 34, 40, 41, 51] (Figure 1). Most members of this family have six predicted transmembrane domains (TMDs) and are predicted to have cytoplasmic amino and carboxy termini (Figure 1). In addition, ZnTs characteristically harbour a long histidine-rich loop between TMD IV and V [(HX_*n*_, *n* = 3 to 6)] and, similar to the ZIPs, place this potential zinc-binding site in the cytosol [52]. An exception to this rule is the human ZnT5 transporter which is predicted to contain 15 TMDs [53] and ZnT6 which harbours a serine-rich loop between TMDs IV and V [54]. Most ZnTs form homodimers with the exception of ZnT5 and ZnT6 which interact to form heterodimers to transport zinc into the early secretory pathway [55]. ZnT proteins have been identified in intracellular compartments that are usually associated with endosomes, Golgi apparatus, or the endoplasmic reticulum (Table 1) [56]. The tissue-specific expression of the ZnTs is varied with the exception of ZnT8 which is expressed predominately in insulin-producing beta cells of the pancreas [57, 58].

## 3. Modes of Zinc Signaling, Zinc Transporters, and Insulin Signaling 

Zinc transporters typically act as zinc sensors, responding to zinc availability to maintain intracellular zinc homeostasis. The mode in which cellular homeostasis is achieved through zinc and zinc transporters is complex and comprehensive, and there are many significant reviews on these processes [3, 26, 30, 33, 34, 38–40, 50, 56, 104]. Accordingly, the following section aims to briefly introduce a number of important concepts by which zinc and zinc transporters maintain cellular homeostasis and to highlight, where possible, the links between zinc transporter modulation and insulin signaling pathways. 

### 3.1. Modes of Zinc Signaling

**Figure 2:**
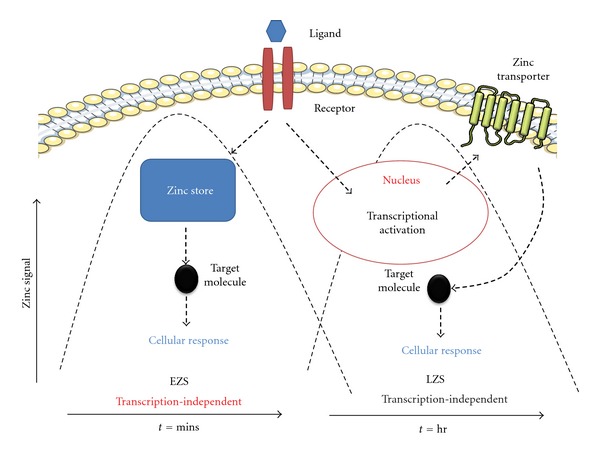
*Early zinc signaling (EZS) and late zinc signaling (LZS)*. EZS involves transcription-independent mechanisms where an extracellular stimulus directly induces an increase in zinc levels within several minutes by releasing zinc from intracellular stores (e.g., endoplasmic reticulum). LSZ is induced several hours after an external stimulus and is dependent on transcriptional changes in zinc transporter expression. Components of this figure were produced using Servier Medical Art, http://www.servier.com/ and adapted from Fukada et al. [30].

Given the large array of zinc transporters that are dedicated to controlling zinc homeostasis (Table 1), it is not surprising that this cation is quickly taking precedence as a leading signaling molecule analogous to calcium. In this context two modes of zinc signaling have been described: “early zinc signaling” (EZS) and “late zinc signaling” (LZS) [105, 106] (Figure 2). EZS involves a rapid change in intracellular levels of free zinc that occurs in minutes due to an extracellular stimulus that is transcription independent [30]. This was revealed in studies by Yamasaki et al. [105] who reported in mast cells a rapid increase in intracellular free zinc from the perinuclear region that includes the endoplasmic reticulum (ER) within minutes following extracellular stimulus with the high affinity IgE receptor (Fc*ε*RI). These authors described this phenomenon as a “zinc wave” that is dependent on both calcium influx and MEK signaling, although calcium influx alone was not sufficient to induce the zinc wave while MEK activation was essential. LZS is also triggered by an extracellular signal and involves transcriptional-dependent changes in expression of proteins implicated in zinc homeostasis such as storage proteins or transporters [30, 106]. Both EZS and LZS modulate numerous cellular processes involved in cell differentiation, proliferation, and growth [35]. The importance of zinc for cell growth and proliferation is well recognized, and deficiencies in zinc cause growth retardation in all organisms investigated [35]. 

In 1980, Coulston and Dandona [107] discovered that zinc exerted a potent stimulatory effect upon lipogenesis in rat adipocytes independent of and additive to that of insulin. These findings suggested that the effects of this cation may have physiological relevance in controlling insulin signaling pathways since zinc is essential for the crystallization of insulin in hexameric complexes [10, 41] and is cosecreted with insulin on exposure to high glucose [41]. Similarly, May and Contoreggi [108], utilizing supraphysiological concentrations (250–1000 *μ*M) of ZnCl_2_, revealed a role for this cation in stimulating glucose transport and oxidation, incorporation of glucose carbon into glyceride-glycerol and glyceride-fatty acid, and inhibition of ritodrine-stimulated lipolysis in rat adipocytes. Equally, Ezaki [109] showed that rat adipocyte cells treated with ZnSO_4_ for 30 minutes stimulated cAMP phosphodiesterase and glucose transporter translocation from the intracellular region to the plasma membrane. Moreover, these authors proposed that this process was not dependent on insulin receptor stimulated kinase activity. This was in contrast to studies by Tang and Shay [110] who demonstrated that treatment of 3T3-L1 adipocytes over 5–10 minutes with ZnCl_2_ increased tyrosine phosphorylation of the IR-*β* subunit of the insulin receptor and enhanced the transport of glucose in the absence of insulin through the PI-3-kinase signal transduction pathway. These studies have since been further delineated with investigations into the role of zinc as an inhibitor of protein tyrosine phosphatases [51, 105, 111]. In fact, inhibition of protein tyrosine phosphatase 1B (PTP1B, a negative regulator of insulin signaling) activity ameliorates high-fat-diet-induced insulin resistance and lipid disorders in mice [112]. Furthermore, mice with a genetic ablation of PTP1B or pharmacological inhibition of its expression and activity are lean and have increased insulin sensitivity [113] and can restore insulin sensitivity in the liver of insulin receptor substrate 2-deficient mice [114]. 

Several groups have examined the mechanisms of the insulin-mimetic activity of zinc on glucose [108, 110, 115–119] and lipid [107, 119] metabolism. Cumulative evidence has revealed zinc as a direct signaling molecule implicated in extracellular signal recognition [105], second messenger metabolism [120], protein kinase activity [110], protein phosphorylation [119, 121], and the modulation of transcription factors [122] and highlights zinc's dynamic role as a “cellular second messenger” in the control of insulin signaling and glucose homeostasis [33, 105, 123]. Insulin is a critically important anabolic hormone implicated in maintaining normal physiological blood glucose. Zinc mediates these effects in part through the inhibition of protein tyrosine phosphatases which increase the net phosphorylation of the insulin receptor and activate its signaling cascade [51, 111]. Accordingly, the effects of zinc on cellular homeostasis appear to be numerous and include the stimulation of glucose uptake and lipogenesis in adipocytes [110], tyrosine phosphorylation of the insulin/IGF-1 receptor and insulin receptor substrate-1 [51, 111, 121], activation of epidermal growth factor receptor [72, 121], inhibition of protein tyrosine phosphatase (PTP) [33, 111], and subsequent activation of mitogen-activated protein kinases (MAPKs) including extracellular-signal-regulated kinases 1 and 2 (ERK1/2), c-Jun N-terminal kinase (JNK) and p38 [20], and an increase in glycogen synthesis through the inhibition of glycogen synthase kinase-3 [115]. 

Several potential mechanisms have been suggested for the role of zinc affecting insulin action including the modulation of insulin receptor tyrosine kinase activity and subsequent defects in insulin-stimulated muscle glycogen synthesis [124, 125]. This raises the prospect that zinc plays a previously unidentified role as a novel second messenger that augments insulin activity. This concept raises a whole new area of research into the pathophysiology of insulin resistance and introduces a new class of drug target with utility for diabetes pharmacotherapy. However, the mechanistic details of how insulin affects intracellular zinc transport and the subsequent signaling cascades associated with glucose homeostasis and insulin resistance are yet to be defined. 

### 3.2. Zinc and Zinc Transporters

The expression and cellular distribution of ZIPS and ZnTs are predominately (but not always) regulated by changes in extracellular and intracellular zinc concentrations [50]. The first study to delineate a role for zinc transporters and zinc homeostasis was performed in 1995 when Palmiter and Findley [49] isolated ZnT1 from a rat kidney cDNA expression library. These authors revealed that transfection of rat ZnT1 cDNA conferred resistance to high levels of extracellular zinc in the zinc-sensitive baby hamster kidney (BHK) cell line. Moreover, ZnT1 overexpression in the BHK cells increased ^65^Zn efflux and reduced the intracellular steady-state concentration of zinc. Following these studies, in 1996 the second ZnT was isolated (ZnT2) (Palmiter et al. [84]). Similar to ZnT1, overexpression of rat ZnT2 cDNA conferred zinc resistance to BHK cells. However, unlike ZnT1 which is located in the plasma membrane and lowers cellular zinc by stimulating cellular zinc efflux, ZnT2 is localized on vesicles and accumulates high concentrations of zinc in the endosomal/lysosomal compartment. ZnT2 is also associated with the inner mitochondrial membrane in mouse MEC mammary cells [126], and attenuation of ZnT2 mRNA expression reduced mitochondrial zinc uptake and mitochondrial zinc pools. 

In studies on rats fed a diet low in zinc (<1 mg Zn/Kg), low expression of ZnT2 was observed in the kidney and small intestine in comparison to rats fed an adequate intake of dietary zinc (30 mg Zn/Kg) [127]. Likewise, ZnT1 and ZnT2 were markedly increased in these tissues when supplied with greater concentrations of dietary zinc (180 mg Zn/Kg) while ZnT4 was refractory to changes in zinc uptake. These findings suggested that a metal-responsive mode of regulation for at least ZnT1 and ZnT2 will involve metal-responsive elements (MREs) in the promoters of these genes. Indeed, the induction of the MTs by metals is mediated by several MREs located in the promoter region of MT genes that recruits the metal response-element transcriptional factor (MTF-1) and is responsible for its transcriptional regulation [128]. Studies in mouse embryo fibroblasts with homozygous deletions of the MTF-1 gene revealed that this protein was essential for basal and zinc-responsive regulation of ZnT1 [129]. MREs have been identified in the ZnT1 promoter [49], and ZnT5 has multiple metal-responsive elements (MREs) in its promoter region that mediate zinc-induced transcriptional activation [40, 130]. 

Many ZnT and ZIP transporters are also regulated by hormones or cytokines (see Lichten and Cousins [38] for a comprehensive review). In LNCaP and PC-3 prostate cancer cells, ZIP1 is regulated by testosterone and prolactin and is consistent with the rapid cellular accumulation and uptake of zinc in these cells [131]. ZIP6 and ZIP14 mRNA expression respond to estrogen stimulation and have reduced response to tamoxifen or fulvestrant [132]. ZIP8 is strongly induced by TNF-*α* in primary human lung epithelial cells [133, 134] and A459 lung cancer cells [134] concomitant with an increase in intracellular zinc. Recent evidence suggests that the mechanism of TNF-*α* induction of ZIP8 is through the NF-*κ*B pathway [134]. ZIP14 is upregulated by IL-6 (but not by TNF-*α*) in mouse hepatocytes where it is suggested to play a role in hypozincemia that accompanies the acute phase response to inflammation and infection [135]. 

Some transporters alter their transcriptional and posttranscriptional expression in response to zinc fluctuations, while others alter their subcellular localization [33]. For example, ZnT1 is predominately localized to the plasma membrane but has recently been shown to locate to the endoplasmic reticulum complexed with EVER transmembrane channel-like proteins in human keratinocytes [136]. EVER and ZnT1 complexes influence intracellular zinc concentration and downregulate transcription factors stimulated by zinc (MTF-1) or cytokines (c-Jun and Elk). Similarly, Kim et al. [137] revealed that the transfection of human embryonic kidney (HEK293) cells with mZIP4 resulted in the localization of this transporter to cytoplasmic vesicles in the perinuclear region. Subsequent depletion of zinc from the HEK293 cells following treatment with the heavy metal chelator TPEN resulted in a redistribution of mZIP4 towards the plasma membrane. Equally, mZIP1 and mZIP3 were found to transit between the plasma membrane and intracellular compartments in zinc-depleted HEK293 cells [60].

ZnT5 is located in the Golgi apparatus and facilitates the transport of zinc into the Golgi lumen for storage. Overexpression of ZnT5 in HeLa cells facilitates zinc uptake into intracellular vesicles suggesting that ZnT5 is capable of accumulating zinc in intracellular compartments [38]. Importantly, ZnT5 is thought to be responsible for loading zinc to secretory, membrane-bound, or organelle-resident proteins to facilitate biological activity of proteins [138]. An important component of ZnT5 as a Golgi zinc influx transporter is its critical role in counteracting the effects of Zip7 to maintain the balance of zinc mobilization between the Golgi and cytosol. 

ZnT4 and ZnT6 have been shown to traffic from the trans-Golgi network to the cytoplasmic vesicular compartment with increasing concentrations of extracellular zinc in rat kidney cells [54]. In transient transfection experiments, Milon et al. [139] revealed that human ZIP1 (hZIP1) has vesicular localization in COS-7 cells. In contrast, transfection of hZIP1 into K562 cells showed plasma membrane localization and suggests that the localization for this protein is under the influence of zinc-responsive regulation. In HEK293 kidney cells grown in zinc-replete medium, mouse ZIP1 and ZIP3 (mZIP) were localized to intracellular organelles [60]; however in the absence of zinc, these proteins were preferentially localized to the plasma membrane suggesting that they are modulated at the posttranslational level to control zinc homeostasis [60]. 

Although many zinc transporters respond to fluctuating zinc levels and alter their subcellular localization, ZIP7 is an exception and is restricted constitutively to the membrane of the Golgi apparatus and endoplasmic reticulum [34]. Studies in human cells including lung fibroblasts (WI-38), prostate epithelial cells (RWPE1), erythroleukemia cells (K-562), and mammary gland epithelial cells (MCF-7) showed ZIP7 antibody fluorescence staining in the perinuclear region of the Golgi apparatus [71]. This was confirmed by treating MCF cells with brefeldin A, a fungal macrocyclic known to disrupt the Golgi apparatus, prior to immunofluorescent staining [71]. Furthermore, ZIP7 gene expression and intracellular location are not altered in response to changes in intracellular zinc status [71]. In this context, it is unlikely that ZIP7 is upregulated by cytosolic zinc concentrations. In contrast, ZIP7 has been shown to be regulated at the post-translational level by suppression of ZIP7 protein translation in response to high zinc concentrations [38, 71]. ZIP7 is involved in the release of zinc from intracellular stores and activation of multiple tyrosine kinases through zinc-mediated inactivation of protein phosphatases [20]. The presence of multiple MAPK-binding motifs and phosphorylation sites on the cytoplasmic domain of ZIP7 suggests that zinc transport by this protein is regulated by phosphorylation [20]. In a recent study, the phosphorylation of the endoplasmic reticulum ZIP7 by protein kinase CK2 was associated with the gated release of zinc from intracellular stores leading to activation of tyrosine kinases and the phosphorylation of AKT and extracellular signaling kinases 1 and 2 [140]. This raises an interesting mechanism for the regulation of ZIP7 through the activity of CK2 possibly through insulin-mediated signaling pathways. Interestingly, treatment of pancreatic beta cells with 100 nM insulin over 4 hours resulted in a significant increase in CK2 kinase activity [141]; however the complete physiological importance of this effect is still to be elucidated. 

It has been suggested that increased expression of ZIP7 could prolong growth factor signaling and contribute to dysfunctional regulatory mechanisms in a number of disease states characterized by the increased activation of tyrosine kinases [20]. Equally decreased expression of ZIP7 might contribute to a deactivation of signaling events associated with cellular signaling through constitutively activated protein phosphatases. Although there are several studies implicating increased protein-tyrosine phosphatase activity as contributing to insulin resistance [51, 110, 111, 113, 114, 121, 142–145], the evidence linking insulin signaling to zinc transport mechanisms and zinc-mediated signaling events is lacking.

### 3.3. Zinc Transporters and Zinc Signaling in T2DM

The interaction between zinc homeostasis and T2DM has been examined based on the insulinomimetic effect of zinc on insulin signaling. Our recent understanding of T2DM and the role that zinc signaling plays in maintaining cellular homeostasis has prompted intensive research efforts into dysregulated cellular zinc partitioning in this chronic disease. Given that health issues associated with T2DM have global significance, there is considerable interest in elucidating the molecular mechanisms responsible for insulin resistance and defects in glycaemic control. Zinc has an integral role in the processing, storage, secretion, and action of insulin in response to changes in elevated glucose concentrations [33, 118]. In vivo, dietary zinc supplements given to *db/db* mice attenuated hyperglycemia and hyperinsulinemia and elevated pancreatic zinc concentrations [117]. Moreover, zinc treatment significantly improved glucose clearance in genetically nonobese IDE-deficient diabetic rats and mice with concomitant diabetes and obesity [123, 146]. In fact, zinc-deficient animals are less sensitive to insulin [147], and oral administration of zinc in animals and humans improves glycaemic control in type 1 and type 2 diabetic patients [3]. In humans, Jansen et al. [9] found a significant reduction in plasma zinc levels in type 1 diabetes and T2DM patients compared to healthy controls and suggest that oral supplementation of zinc may qualify as a potential adjunct therapy in T2DM patients by promoting insulin signaling. A recent systemic review of the literature and meta-analysis on 25 studies reporting the effects of zinc supplementation on T2DM revealed that those patients receiving the zinc therapy had improved glycaemic control and health lipid parameters [148]. 

In the context of zinc transporter systems the most well studied in diabetes is ZnT8. In human type 1 diabetes, ZnT8 was targeted by autoantibodies in 60%–80% of new onset cases [149]. Recently ZnT8 is purported to have a role in T2DM. The expression of ZnT8 is predominately within the insulin-producing beta cells of the pancreas [57] but has also been identified at much lower levels in other tissues such as kidney and testis [42]. ZnT8 overexpression in INS-1 cells enhanced glucose-stimulated insulin secretion [57] and the downregulation of ZnT8 in this system showed reduced insulin content and secretion in response to a hyperglycaemic stimulus [150]. Mice with a targeted beta cell ZnT8 “knock out” display deficiencies in glucose intolerance [151]. Moreover, ZnT8 null mice display diet-dependent abnormalities in glucose tolerance [152], insulin secretion [152, 153], and body weight [152, 154]. 

Several genomewide association studies (GWASs) have implicated ZnT8 in T2DM in humans [8, 155–157]. Thus, having two copies of the at-risk polymorphic variant rs13266634, a nonsynonymous single nucleotide polymorphism (SNP) (Arg325Trp) in ZnT8 is associated with a 43% increase risk of developing diabetes [158]. Recently a GWAS study identified variants in ZnT8 to be associated with a greater risk for T2DM from 1508 Chinese Han T2DM patients and 1500 age- and gender-matched healthy controls [156]. All subjects were genotyped for 3 tagging SNPs (rs2466295, rs4876703, rs11558471). The genotype and the allele distributions of the AA genotype of rs11558471 were more common in the T2DM subjects than the control group [156]. The frequency of the A-C-A haplotype was significantly greater in the T2DM subjects compared to the controls while the A-G-A haplotype was significantly reduced in the T2DM subjects compared to the controls. These authors predict that the A-C-A haplotype is a risk factor for T2DM while the A-G-A haplotype is protective for T2DM in Chinese Han people [156]. Accordingly, it is suggested that polymorphisms in ZnT8 affect insulin secretion and increase the risk for T2DM as binding of zinc is paramount for the crystallization of insulin [151, 152].

Several other zinc transporters, although not directly associated with diabetes, are implicated in insulin-mediated signaling and glycaemic control in animal models and humans. Huang et al. [159] demonstrated that overexpression of ZnT7 in pancreatic insulinoma RIN5mf cells increased insulin secretion upon glucose stimulation. Following these studies Huang et al. [160] revealed that ZnT7 knockout mice were more susceptible to diet-induced glucose intolerance and insulin resistance, and this was concomitant with a reduction in the mRNA expression of the insulin receptor, insulin receptor substrate 2, and Akt1 in primary skeletal myotubes. ZnT3, ZnT5, and ZnT8 gene expression is differentially regulated by glucose in INS-1E cells and ZnT3 knockdown decreased insulin gene expression and secretion and resulted in hyperglycemia in streptozotocin-treated ZnT3 null mice [161]. Similarly, elevated glucose concentrations increased free cytosolic zinc in mouse pancreatic islets and were associated with an increase in the mRNA expression of ZIP6-8 [162]. These authors suggested that glucose induces cytosolic zinc leading to the processing and storage of insulin and associated increase in the ZIP importers. 

The studies presented on the role of zinc and zinc transporters in cell-based and rodent knockout and/or overexpression systems and allelic variants in humans suggest that this family of proteins plays a key role in the pathogenesis of diabetes. Identifying how zinc and zinc transporters play a role in insulin signaling and glycaemic control will be of upmost importance in elucidating novel therapeutic options for the treatment and prevention of T2DM. 

## 4. Conclusions and Perspectives 

Zinc is an essential trace metal that is implicated in many physiological and metabolic processes. The ubiquitous nature of zinc in physiological systems suggests that atypical levels are likely to have many biological and clinical effects. The true significance of zinc in cellular signaling is just emerging. In this context the zinc transporters play an essential role in insulin and glucose homeostasis. The inhibition of protein tyrosine phosphatases by zinc under physiological conditions involving zinc transporter mechanisms has widespread implications for understanding insulin resistance and disease progression. While a clear role for ZnT8 in insulin production and susceptibility to T2DM has been established, in the future, confirmation of a role for zinc transporters and zinc signaling in insulin activity will establish zinc homeostasis as a key element in the pathogenesis of T2DM. 

## Figures and Tables

**Table 1:** Zinc transporters, cellular location, tissue-specific expression, and disease associations.

Zinc transporter	Cellular localization	Tissue expression	Disease associations	References
SLC39A1/ZIP1	Plasma membrane	Ubiquitously expressed	Prostate cancer	[59–61]
SLC39A2/ZIP2	Plasma membrane	Blood, prostate	Carotid artery disease	[62–64]
SLC39A3/ZIP3	Plasma membrane, intracellular compartments	Mammary gland, prostate	Unknown	[60, 65]
SLC39A4/ZIP4	Apical membranes	Small intestine, stomach, colon, kidney, brain	Pancreatic cancer, acrodermatitis enteropathica (AE)	[62, 66, 67]
SLC39A5/ZIP5	Basolateral membranes	Pancreas, kidney, liver, spleen, colon, stomach	Unknown	[68, 69]
SLC39A6/ZIP6	Plasma membrane	Ubiquitously expressed	Breast cancer	[70]
SLC39A7/ZIP7	Golgi apparatus, endoplasmic reticulum	Ubiquitously expressed	Breast cancer	[71, 72]
SLC39A8/ZIP8	Vesicles	Ubiquitously expressed	Unknown	[73, 74]
SLC39A9/ZIP9	Trans-Golgi network	Ubiquitously expressed	Unknown	[75]
SLC39A10/ZIP10	Plasma membrane	Ubiquitously expressed	Breast cancer	[76, 77]
SLC39A11/ZIP11	Unknown	Mammary gland	Unknown	[78]
SLC39A12/ZIP12	Unknown	Retina, brain, testis, lung	Schizophrenia	[79]
SLC39A13/ZIP13	Golgi apparatus	Ubiquitously expressed	Ehlers-Danlos syndrome	[80, 81]
SLC39A14/Zip14	Plasma membrane	Ubiquitously expressed	Asthma	[82, 83]
SLC30A1/ZnT1	Plasma membrane	Ubiquitously expressed	Alzheimer's disease, Pancreatic cancer	[19, 25, 49]
SLC30A2/ZnT2	Vesicles, lysosomes	Pancreas, kidney, testis, epithelial cells, small intestine	Unknown	[84]
SLC30A3/ZnT3	Synaptic vesicles	Brain, testis	Alzheimer's disease	[85, 86]
SLC30A4/ZnT4	Intracellular compartments	Mammary gland, brain, small intestine, placenta, blood, epithelial cells	Alzheimer's disease	[25, 87]
SLC30A5/ZnT5	Secretory vesicles, Golgi apparatus	Ubiquitously expressed	Osteopenia	[53, 88]
SLC30A6/ZnT6	Secretory vesicles, Golgi apparatus	Small intestine, liver, brain, adipose tissue	Alzheimer's disease	[25, 54]
SLC30A7/ZnT7	Golgi apparatus	Retina, small intestine, liver, blood, epithelial cells, spleen	Prostate cancer	[89, 90]
SLC30A8/ZnT8	Secretory vesicles	Pancreatic B-cells	Type 1 and 2 diabetes mellitus	[57, 91, 92]
SLC30A9/ZnT9	Cytoplasm, nucleus	Ubiquitously expressed	Unknown	[93]
SLC30A10/ZnT10	Unknown	Liver, brain	Parkinson's disease, dystonia, liver disease	[94–96]
MT1	Cytoplasm, nucleus, mitochondria	Ubiquitously expressed	Unknown	[97, 98]
MT2	Cytoplasm, nucleus, mitochondria	Ubiquitously expressed	Unknown	[99, 100]
MT3	Cytoplasm, nucleus	Brain, testis	Alzheimer's disease	[101]
MT4	Cytoplasm, nucleus	Squamous epithelia	Unknown	[102, 103]
